# Photodynamic therapy upregulates expression of HIF-1α and PD-L1 in related pathways and its clinical relevance in non-small-cell lung cancer

**DOI:** 10.1186/s40001-024-01780-0

**Published:** 2024-04-12

**Authors:** Wen Sun, Yuan Cheng, Xiaoyu Ma, Zhou Jin, Qi Zhang, Guangfa Wang

**Affiliations:** 1 Department of Respiratory and Critical Care Medicine, Peking University First Hospital, Beijing, 100034 China; 2 Department of Pulmonary and Critical Care Medicine, Peking University First Hospital, No. 8 Xishiku Street, Xicheng District, Beijing, 100034 China

**Keywords:** Photodynamic therapy (PDT), Non-small cell lung cancer (NSCLC), Hypoxia inducible factor 1α (HIF-1α), Programmed cell death 1 ligand 1 (PD-L1), Immune checkpoint inhibitors (ICIs)

## Abstract

**Background:**

Photodynamic therapy (PDT) is a promising interventional treatment approach that contributes to antitumor immunity. It has been reported that PDT can enhance the effectiveness of immune checkpoint inhibitors (ICIs), but its mechanism is yet unclear. Herein, we implemented bioinformatics analysis to detect common pathways and potential biomarkers in non-small cell lung cancer (NSCLC), PDT, and NSCLC immunotherapy to investigate potential links between PDT, immunotherapy and NSCLC, and their clinical impact.

**Methods:**

Differentially expressed genes in NSCLC- and NSCLC immunotherapy-related data in the GEO database were intersected with PDT-related genes in the GeneCards database to obtain candidate genes and shared pathways. Enrichment analysis and protein–protein interaction were established to identify key genes in functionally enriched pathways. The expression profiles and the prognostic significance of key genes were depicted.

**Results:**

Bioinformatics analysis showed that HIF-1α was screened as a prognostic gene in hypoxia, HIF-1, and PD-L1-related signaling pathways, which was associated with clinical response in NSCLC patients after PDT and immunotherapy. In vivo experiments showed that PDT could inhibit tumor growth and upregulate HIF-1α and PD-L1 expressions in NSCLC tissues with a positive correlation, which might influence the blocking activity of ICIs on the HIF-1, and PD-L1-related signaling pathways.

**Conclusions:**

PDT might improve the clinical response of ICIs by upregulating tumor HIF-1α and PD-L1 expressions in NSCLC.

## Introduction

As a first-line treatment, immune checkpoint inhibitors (ICIs) can induce substantial therapeutic responses in advanced non-small cell lung cancer (NSCLC) [1]. It is possible, however, that patients may not respond to ICIs or may develop drug resistance [1]. Thus, enhancing immunotherapy efficacy and reversing immune resistance is currently a clinical challenge. Local treatment may increase immunotherapy efficacy, and photodynamic therapy (PDT), a type of interventional locoregional therapy, has the advantage of minimal invasiveness, high tumor selectivity, and mild complications [2]. PDT can regulate immune response in patients with malignant lung diseases by activating innate and adaptive immunity to recognize and kill tumor cells [2–4]. In the age of tumor immunotherapy, it is important for PDT to break traditional barriers and combine with systemic antitumor treatment to achieve a more effective treatment response. Research on new photosensitizers combined with ICIs are currently under preclinical study but may take a long time to be applied in clinical practice [5]. Clinically, a more practical and realistic approach would be to demonstrate the synergistic effectiveness of traditional PDT combined with immunotherapy.

As PD-1/PD-L1 expression plays an important role in tumor immunity, their targeting monoclonal antibodies can restore immune cell activity and induce antitumor immunity [6]. There is evidence that PD-L1 expression is a significant predictor of immunotherapeutic response [7], that is, tumors with higher PD-L1 expression respond better to immunotherapy. Tumor-specific PD-L1 could be induced by HIF-1α in certain circumstances [8]. HIF-1α is reportedly responsible for tumor adaptation to hypoxic microenvironments through the HIF-1 pathway and hypoxia is a hallmark of solid tumors, which regulates the pathophysiology of tumorigenesis, including proliferation, angiogenesis, and metabolism [9–11]. The HIF-1α expression level may be upregulated in PDT through mechanisms of oxidative stress, hypoxia, and reactive oxygen species (ROS) [12–14]. These findings raise an interesting possibility that HIF-1α and hypoxia-related signaling pathways are potential mechanisms governing PD-L1 expression in tumor tissue when using traditional PDT. This could possibly increase the sensitivity of sequential systemic immunotherapy. Herein, we aimed to investigate the interaction of the PDT and tumor HIF-1α/PD-L1 expression to assess their prognostic values.

## Materials and methods

### Bioinformatics analysis methods

#### Data source

**Fig. 1:**
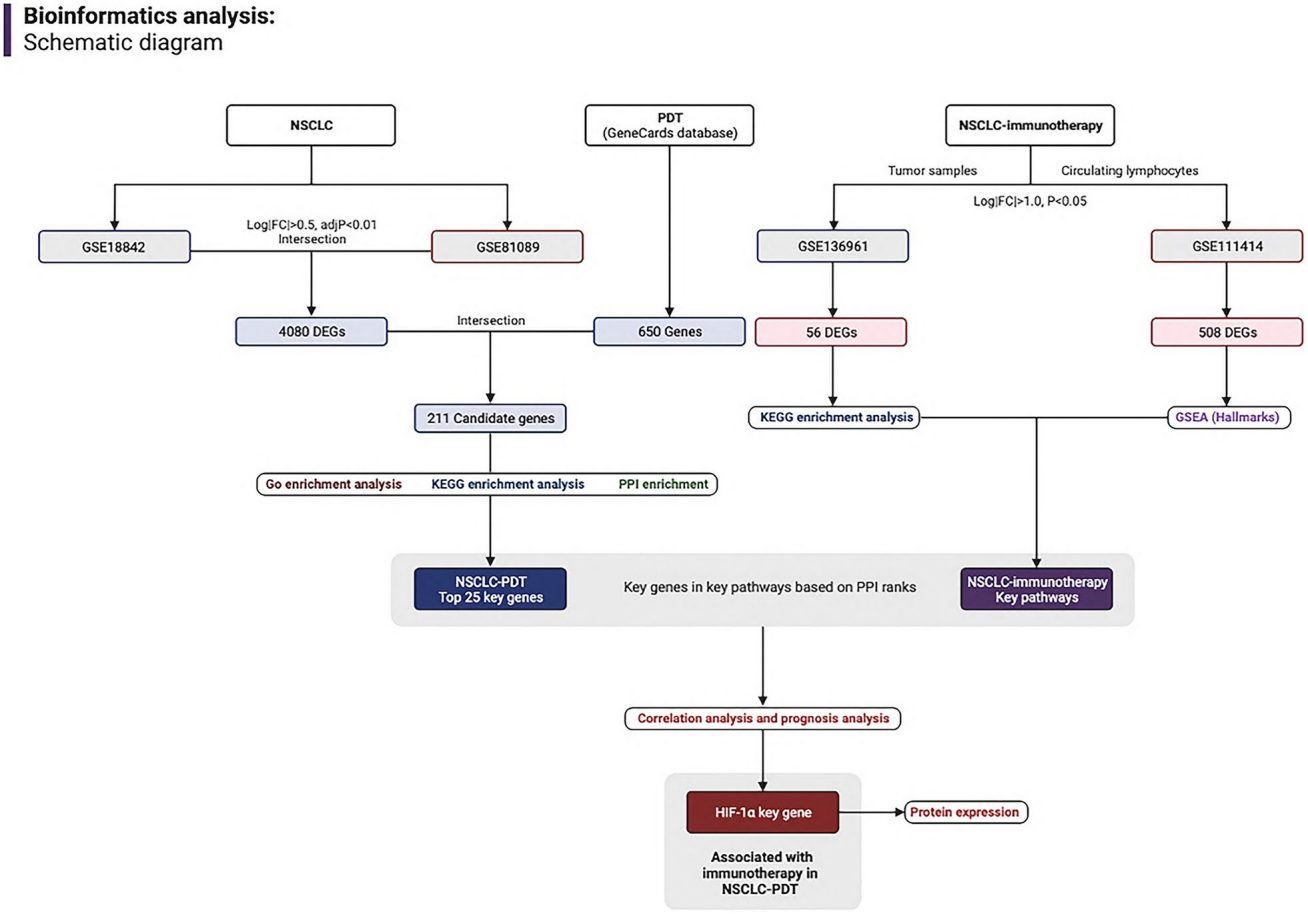
Schematic diagram of bioinformatics analysis related to the NSCLC and its treatment outcomes

The gene expression omnibus (GEO, https://www.ncbi.nlm.nih.gov/gds) matrix was screened for differentially expressed genes (DEGs) associated with the clinical benefit of immunotherapy in patients with NSCLC. Limma and edgeR packages in R software (version 4.1.1) were utilized in DEGs screening (Fig. 1). NSCLC-related datasets GSE18842 and GSE81089 included more than 90 tumor and normal tissues updated in the last four years. NSCLC-immunotherapy datasets GSE136961 and GSE111414 were obtained to evaluate clinical remission and its duration from samples and peripheral blood CD8 + T cells. PDT-related genes from GeneCards database (https://www.genecards.org/) were obtained to select the top 650 genes with the threshold of the median score.

#### Functional enrichment analysis

Gene ontology (GO) and Kyoto Encyclopedia of Genes and Genomes (KEGG) were used to explore the molecular mechanism and crucial pathways using David tool (https://david.ncifcrf.gov/summary.jsp), image GP tool (https://www.bic.ac.cn/ImageGP/index.php/Home/Index/index.html) and “clusterProfiler” package in R. Gene set enrichment analysis (GSEA, HALLMARK, version 4.3.2) were employed with the standard threshold (|NES|> 1, NOM p-value < 0.1, FDR q-value < 0.20). The intersected candidate genes were obtained by jvenn tool (http://jvenn.toulouse.inra.fr/app/example.html) and constructed by String database (https://string-db.org/) with the highest combined score and drawn by Cytoscape (version 3.9.0) and MCODE plugin. UCSC Xena database (http://xena.ucsc.edu/) was utilized for the analysis of correlations between key factors in TCGA Lung Cancer.

#### Genetic mutation and prognostic analysis

The cBioPortal database was used to examine the genetic mutation status in the TCGA cohort. The exploration of survival data in TCGA and GEO database was shown by KM-plotter (http://kmplot.com/analysis/index.php?p=background), and the protein expression data on patients by immunohistochemistry were explored and verified by The Human Protein Atlas database (https://www.proteinatlas.org/). Following that, we identified key genes affecting treatment response in NSCLC patients using PD-1/PD-L1 blockade and receiving PDT (Fig. 1).

### In vivo PDT methods

#### Cell line

The human lung cancer cell A549 was acquired from the American Type Culture Collection, and cultivated in Dulbecco’s modified eagle medium with 10% fetal bovine serum and 1% penicillin–streptomycin in a humid atmosphere with 5% CO_2_ at 37 °C.

#### Animal model

BALB/c-nude mice (4-week, male; Beijing Vital River Laboratory Animal Technology Co., Ltd., China) raised in specific pathogen-free environment in the Animal Science Center of Peking University First Hospital (Beijing, China). A549 cells (1 × 10^7^/ml) with Matrigel were injected subcutaneously above the right anterior armpit of mice. The tumor volume (TV) was measured daily as follows [15]: Volume (mm^3^) = (a × b^2^)/2, with a and b representing the long and short diameters, respectively. This in vivo study was approved by the Ethics Committee of Peking University First Hospital (J202126).

#### PDT procedure

Seven groups of mice were randomly selected (*n* = 78). The mice in PDT group were intravenously treated with 3 mg/kg hematoporphyrin derivative (HPD, Chongqing Milelonge Biopharmaceutical Co., Ltd, Chongqing, China), and superficially irradiated with 100, 200, 300, 400, 500 J/cm^2^ light dose using a 630-nm laser after 48 h injection. The control group was only treated with HPD without irradiation or only received normal saline alone. On day 28, mice were euthanized by carbon dioxide narcosis, and tumors were collected for further study.

#### Western blot

We extracted the total protein in tumor tissues using RIPA protein lysate and measured its content (30 μg) with the BCA protein assay kit. Next, we isolated the protein sample in 4–12% SDS‒PAGE (GenScript-China, China), and transferred it to nitrocellulose membranes, imprinted with 5% nonfat dry milk for 1 h at room temperature, and subsequently incubated with primary antibodies (anti-β-actin, 1:1000, Beijing Zhongshan Golden Bridge Biotech, China; anti-HIF-1α, 1:2000, Abcam; and anti-PD-L1, 1:1000, Abcam) overnight at 4 °C and with corresponding secondary antibodies for 1 h. Then, we visualized the film by enhanced chemiluminescence and quantified it by Image J software.

#### Real-time quantitative reverse transcription PCR (RT-qPCR)

The total RNA of tumor tissues was isolated using TRIzol® reagent (Life Technologies, USA), and converted into cDNA by Transcriptor First Strand cDNA Synthesis Kit (Thermo Fisher Scientific, USA) and amplificated by PowerUp™ SYBR™ Green Master Mix (Applied Biosystems, Thermo Fisher Scientific, USA). Table 1 shows the primer sequences, and Table 2 shows the thermal cycler parameters.

**Table 1:** Primer sequences for quantitative real-time PCR

Gene	Sequences (5ʹ → 3ʹ)	
β-actin	Forward	GCTTCTTTGCAGCTCCTTCGT
	Reverse	AGCGCAGCGATATCGTCATC
PD-L1	Forward	GCTGCACTAATTGTCTATTGGGA
	Reverse	AATTCGCTTGTAGTCGGCACC
HIF-1α	Forward	GAACGTCGAAAAGAAAAGTCTCG
	Reverse	CCTTATCAAGATGCGAACTCACA

**Table 2:** Thermal cycler parameters for quantitative real-time PCR

Initial denaturation	
50 °C	2 min
95 °C	2 min
Amplification cycle (40 cycles)	
95 °C	15 s
60 °C	15 s
72 °C	60 s
Final extension	
60 °C	10 min

### Statistical analysis

SPSS 26.0 (SPSS Inc., Chicago, USA), GraphPad Prism 9.0, and R software were used to analyze and visualize the data. We presented our results as mean ± SD or median ± IQR according to the data distribution. One-way analysis of variance, Kruskal‒Wallis test, Spearman and Pearson correlation were employed to compare relationships between key genes and differences between in vivo groups. P < 0.05 was considered statistically significant.

## Results

### PDT-related candidate genes have a major role in NSCLC

**Fig. 2:**
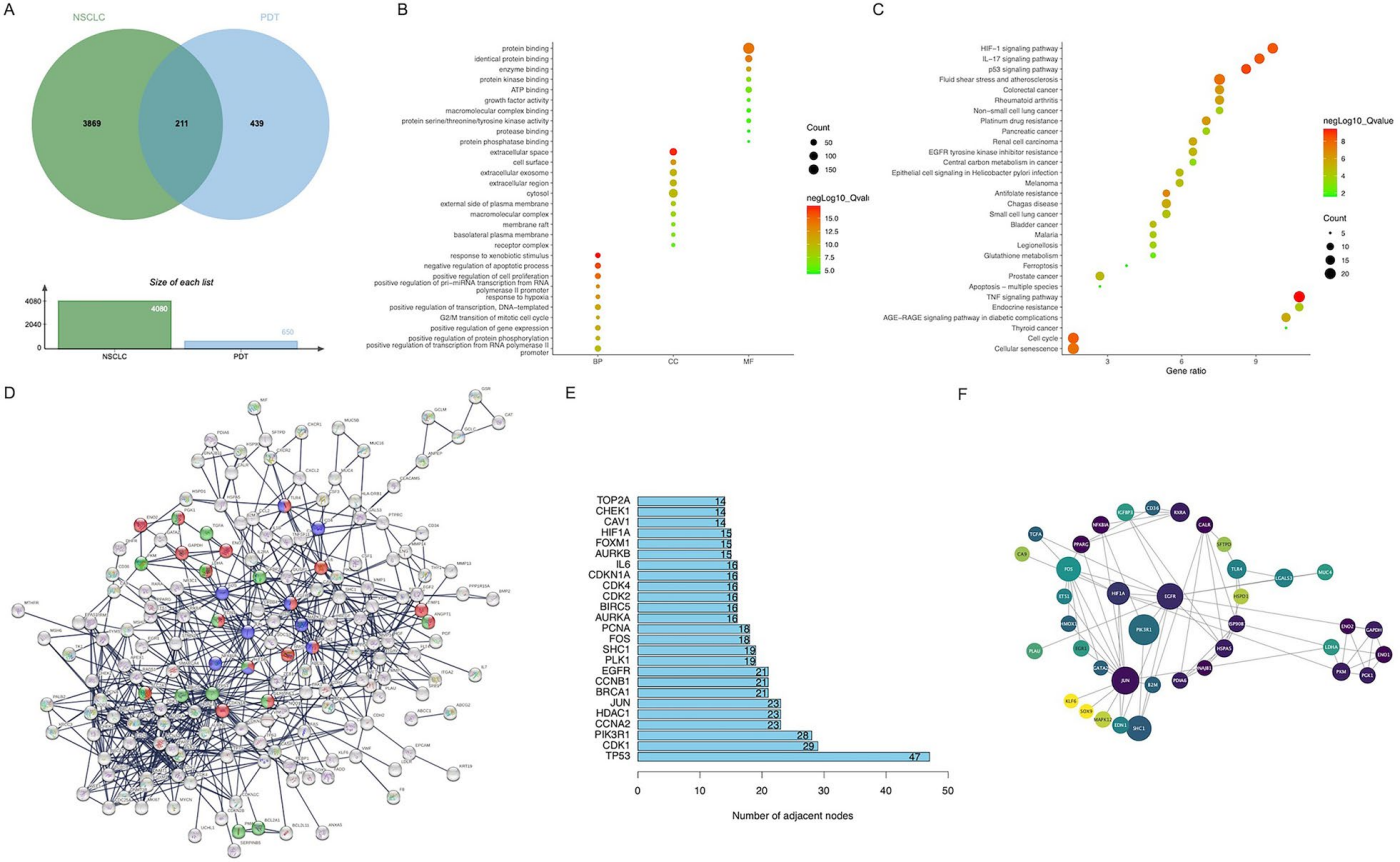
Enrichment analysis and PPI network of candidate genes in NSCLC-PDT database. **A** The Venn diagram of DEGs from datasets from GEO and GeneCards database. **B** Bubble chart of GO enrichment analysis of candidate genes. **C** Bubble chart of KEGG enrichment analysis of candidate genes. **D** PPI network of candidate genes using the STRING database. Red represents HIF-1 pathway-related genes, purple represents PD-L1 and PD-1 checkpoint pathway-related genes, and green represents PDT-induced HIF-1 signaling pathway-related genes. **E** Bar chart of the TOP25 genes based on degree value. **F** The correlation of significant modules in PPI network. The node size was varied by PPI degrees, and the color was different with MCODE scores

We retrieved NSCLC-related datasets (GSE18842 and GSE81089) from GEO database, and PDT-related genes from GeneCards database. GSE18842 contained a total of 7434 DEGs, and GSE81089 contained a total of 9645 DEGs. 211 candidate genes were obtained from the intersection of 650 PDT-related genes with the above NSCLC-related DEGs (Fig. 2A). Based on GO enrichment analysis, 211 candidate genes showed significant effects on response to xenobiotic stimulus (GO:0009410), negative regulation of apoptotic process (GO:0043066), positive regulation of cell proliferation (GO:0008284), and response to hypoxia (GO: 0001666) in the BP, and they were more involved in the CC of extranuclear area and in the MF of protein binding in GO analysis (Fig. 2B). Based on KEGG pathway analysis, the most enriched pathways of 211 candidate genes were TNF pathway (hsa04668), HIF-1 pathway (hsa04066), IL-17 pathway (hsa04657), p53 pathway (hsa04115), and NSCLC pathway (hsa05223) (Fig. 2C). Subsequently, 211 candidate genes were imported into the String database to construct the connected network (209 nodes, 615 edges; p < 0.0001, Fig. 2D). The top 25 key genes were identified based on degree values and displayed using Cytospace (Fig. 2E, F).

### Hypoxia, HIF-1, and PD-L1-related signaling pathways are responsible for PD-1 or PD-L1 blocking clinical responses in NSCLC-PDT

**Fig. 3:**
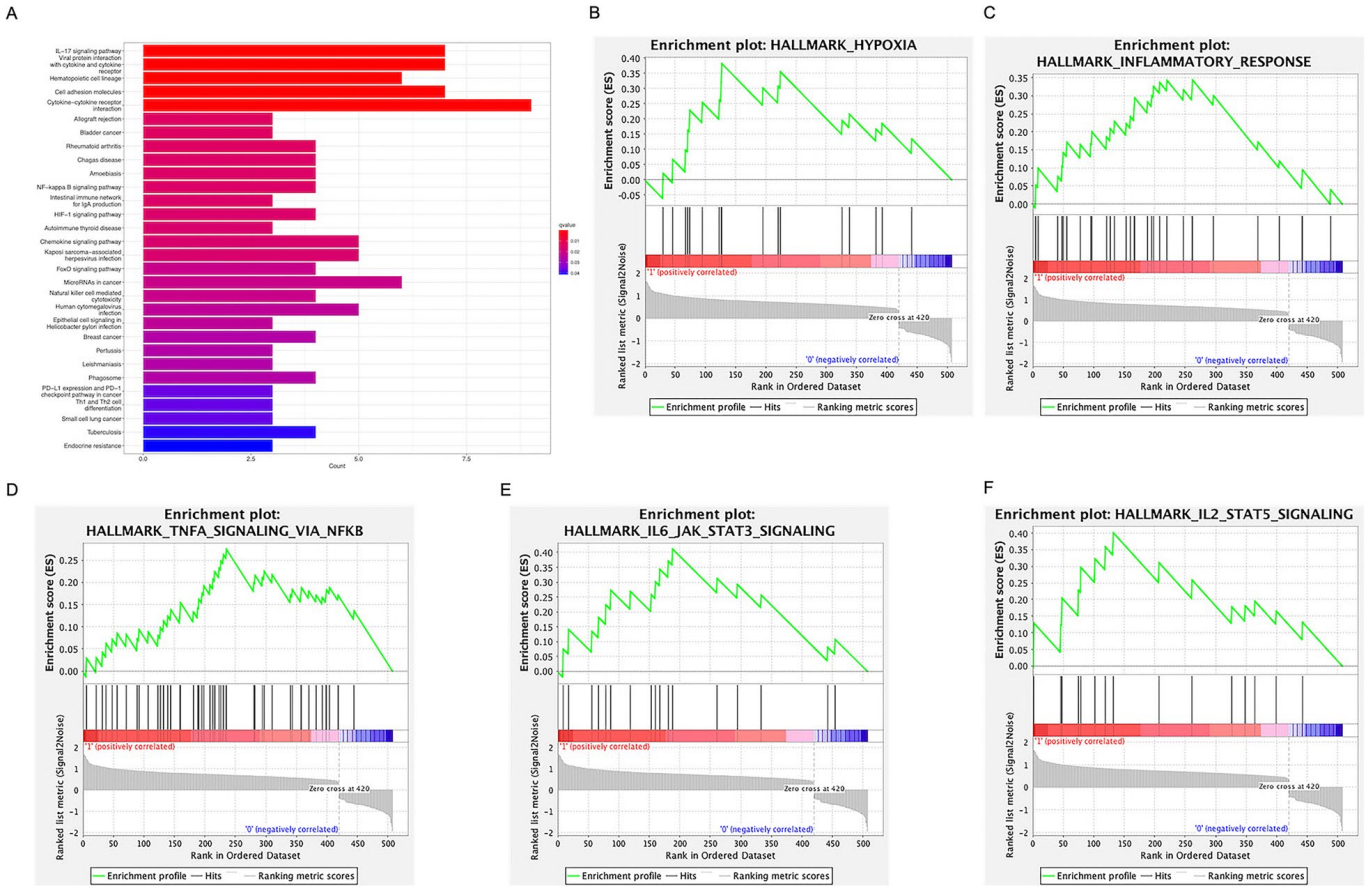
Enrichment analysis of DEGs in NSCLC-immunotherapy database. **A** Bar chart of KEGG analysis in the GSE136961. **B**–**F** GSEA enrichment results of DEGs in the GSE111414

We used KEGG pathway analysis on GSE136961 to explore the immune characteristics of long-term responses of PD-1 or PD-L1 blocking therapy. The results indicated that IL-17 pathway (hsa04657), NF- κB pathway (hsa04064), HIF-1 pathway (hsa04066), FoxO pathway (hsa04068) and PD-L1-related signaling pathways in cancer (hsa05235) were mainly enriched (Fig. 3A). In addition, we utilized GSEA on GSE111414 to investigate the resistance mechanisms to PD-1 or PD-L1 blocking therapy in circulating lymphocytes. The results showed that hypoxia, inflammatory, TNFα, IL-6/STAT3, and IL-2/STAT5 enrichments of CD8^+^ T cells might contribute to PD-1or PD-L1 treatment resistance in NSCLC (Fig. 3B–F). Combining the above functional enrichment analysis results, we identified hypoxia, HIF-1 pathway, and PD-L1-related signaling pathway as critical pathways to PD-1 or PD-L1 treatment responses in NSCLC.

### HIF-1α is the key gene that may affect the prognosis of immunotherapy in NSCLC-PDT

**Fig. 4:**
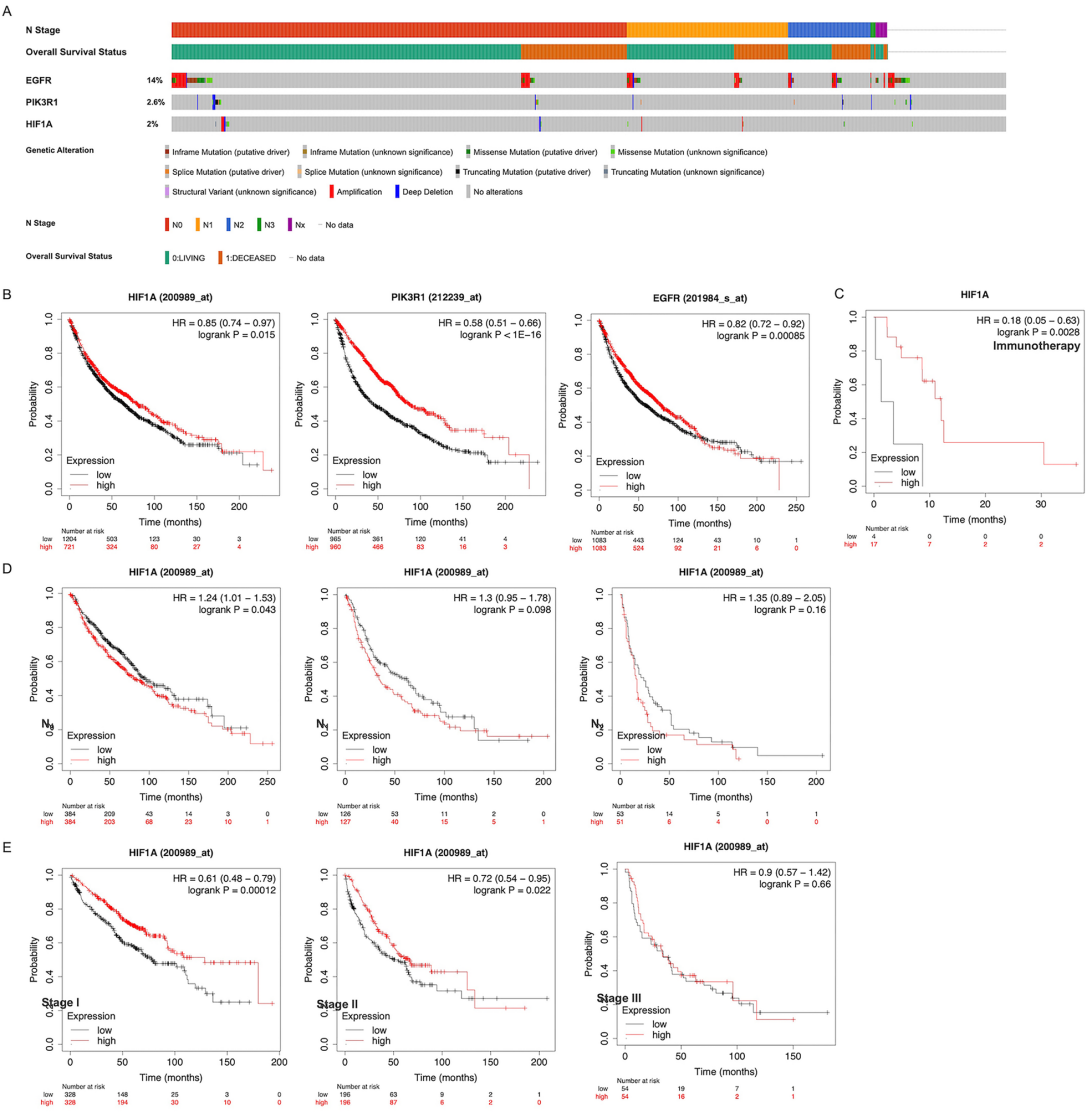
Mutation of key genes expression in tumor tissue of NSCLC patients and their impact on the overall survival. **A** Genetic mutation status of EGFR, PIK3R1, HIF-1α genes in the TCGA cohort in the cBioPortal website (1144 patients). **B** Kaplan–Meier curve and overall survival data of NSCLC patients. **C** The overall survival of NSCLC patients after immunotherapy. **D**, **E** The overall survival curves according to the status of the HIF-1α gene expression and of lymph node metastasis (**D**) and tumor stages (**E**) in NSCLC patients

We obtained 3 key genes (HIF-1α, PIK3R1, EGFR) in the above key pathways based on the PPI ranks. Identifying the extent of inherited mutations contributes to understanding the role of genes in disease development. EGFR had the highest mutation frequency (14%), followed by PIK3R1 (2.6%) and HIF-1α (2%), and main mutation type was amplification for EGFR, missense mutation for HIF-1α, and deep deletion for PIK3R1 (Fig. 4A). HIF-1α, PIK3R1, and EGFR were significantly linked to overall survival in NSCLC (Fig. 4B). A significant difference in prognosis of NSCLC patients treated with immunotherapy was observed between high and low expression level groups of HIF-1α (Fig. 4C), but not in the other 2 genes. Moreover, the clinical significance of HIF-1α in NSCLC varied depending on tumor stage and lymph node involvement (Fig. 4D, E).

**Fig. 5:**
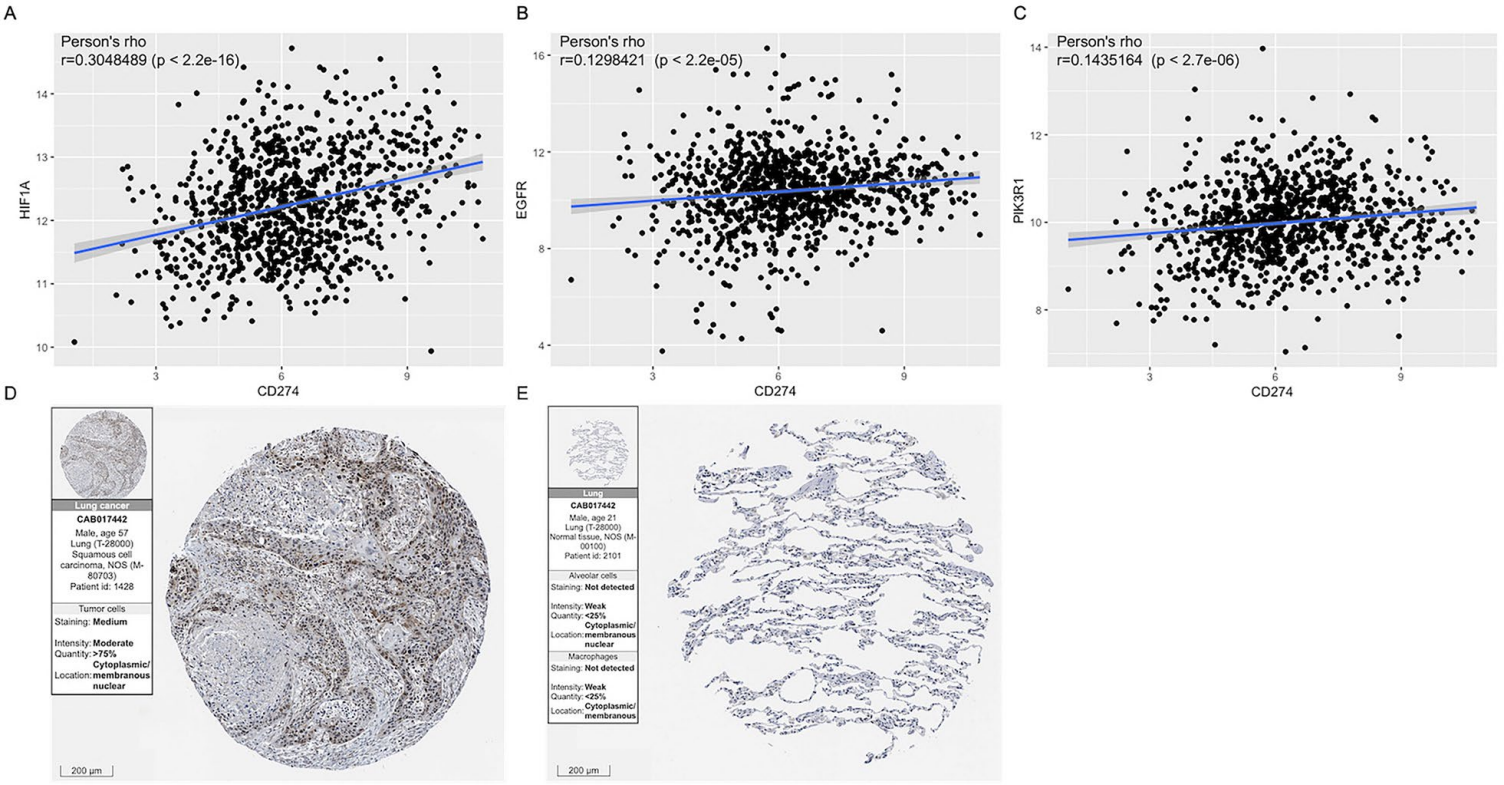
Correlation analysis and immunohistochemistry analysis. **A**–**C** The relationship of PD-L1 and HIF-1α (**A**), EGFR (**B**), and PIK3R1 (**C**) expression in tumor tissue of NSCLC patients in the TCGA cohort (1062 samples). **D**, **E** Immunohistochemistry analysis of HIF-1α in tissue sections (**D**) and normal tissues (**E**) of NSCLC patients in the Human Protein Atlas database

Then, we found a significant correlation between 3 key genes (HIF-1α, EGFR, and PIK3R1) and PD-L1 expression, in which HIF-1α had the strongest correlation (Fig. 5A–C). We further validated HIF-1α protein expression in lung cancer patients and observed a higher HIF-1α expression level in tumor tissue than in normal tissue (Fig. 5D, E). Therefore, it was speculated that PDT might affect HIF-1α and PD-L1 expression by mediating their related signaling pathways in tumors to influence the survival of NSCLC patients after immunotherapy.

### PDT inhibited tumor growth and upregulated the expression level of HIF-1α and PD-L1

**Fig. 6:**
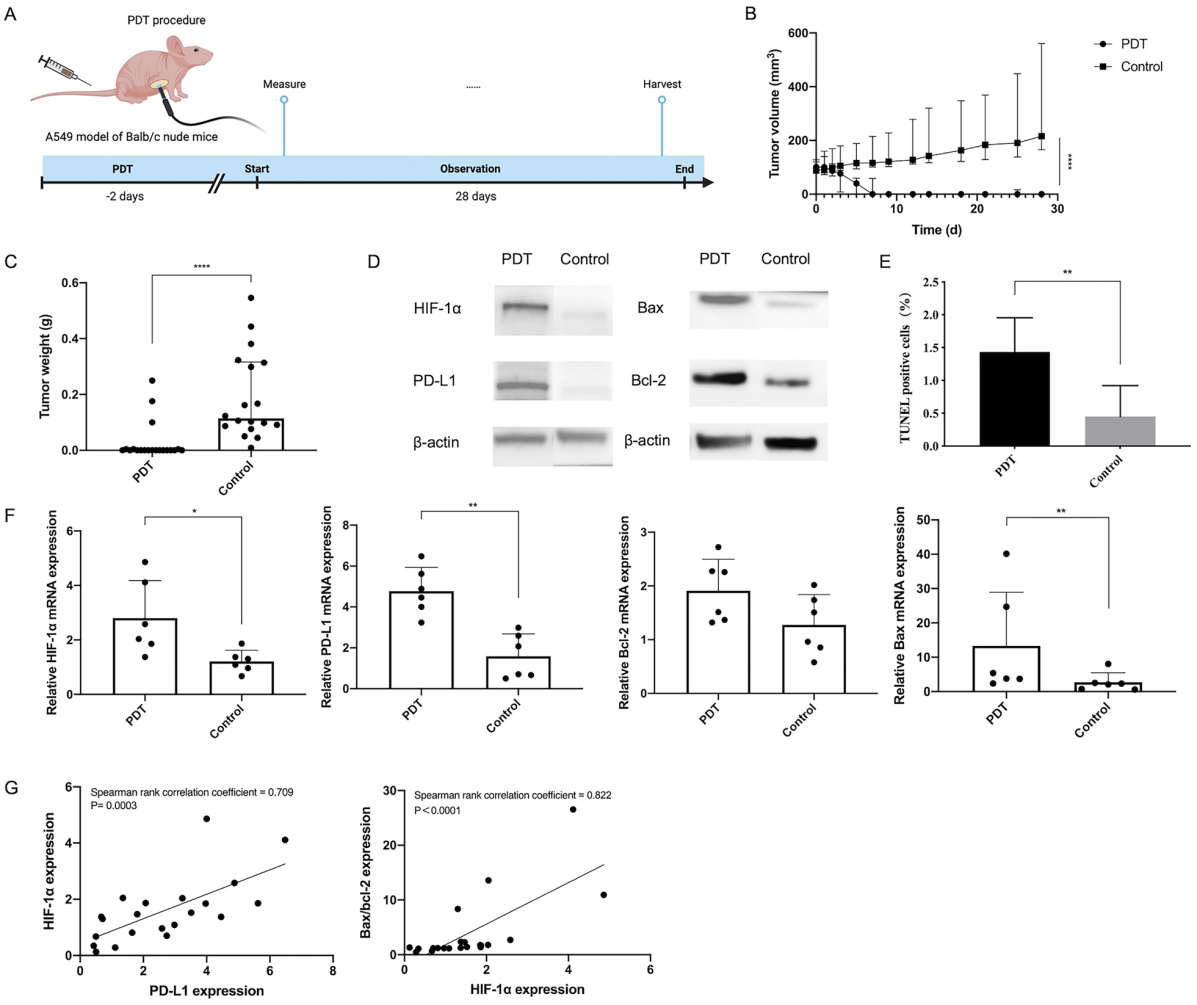
PDT induces antitumor effects and enhances PD-L1 and HIF-1α expression in vivo. **A** Schematic illustration of the experiment design to examine PDT responses. **B** Changes in tumor volume with time after PDT. **C** Tumor weight was tested after being stripped from mice. **D** HIF-1α, PD-L1, Bax and Bcl-2 protein expression by western blot assay in A549 tumors after PDT. **E** TUNEL positive cells in PDT and control group. **F** Relative expression of HIF-1α, PD-L1, Bax, Bcl-2 mRNA levels by real-time PCR in A549 tumors after PDT. **G** Correlation analysis among all the tissue samples of HIF-1α and PD-L1 mRNA expressions, and Bax/Bcl-2 and HIF-1α mRNA expressions. ^*^ *p* < 0.05, ^**^ *p* < 0.01, ^***^P < 0.001, ^*^.^***^p < 0.0001

To further validate the above results, we used A549 cell-inoculated Balb/c nude mice to verify the treatment effect and explore the mechanism of PDT. The tumor growth (volume and weight) of A549-xenograft mice was significantly reduced in treatment groups. The HIF-1α and PD-L1 expressions were significantly increased in PDT groups according to western blot and qRT-PCR results. Thus, we combined mice in the optimal dose group into a single PDT group and re-analyzed data. We then observed that PDT significantly enhanced HIF-1α/PD-L1 expression with positive strong correlation comparing with control groups (Fig. 6). These results indicated that HIF-1 signaling could upregulate the expression of HIF-1α/PD-L1, thus exerting an anti-tumor effect.

### PDT regulated Bax/Bcl-2 expression to promote tumor apoptosis

To investigate downstream target genes in HIF-1 pathway associated with hypoxia-induced apoptosis, we examined the expression level of Bcl-2 family members by qRT-PCR and confirmed the apoptosis level by TUNEL test. The result showed that PDT could induce apoptosis by upregulating Bax and Bcl-2 expression, and Bax/Bcl-2 expression ratio had significantly positive correlation with HIF-1α expression (Fig. 6).

## Discussion

Hypoxia is a distinct hallmark of malignancy, including lung cancer [9]. It can be induced by PDT in the tumor microenvironment via generating ROS and mediating oxidative stress [12, 16, 17]. Both PDT and treatment of hypoxia can activate and stabilize HIF-1α expression, thus regulating the transcription of PD-L1 mRNA and affecting PD-L1 expression [10, 12, 13, 16]. Previous studies on solid tumors demonstrated that HIF-1α blockage could inhibit PD-L1 expression to influence the anti-tumor effect, thus providing a rationale for combination therapies with immunotherapies [18, 19].

As only 20–40% of cancer patients benefit from ICIs targeting PD-1/PD-L1, an awareness of the immune sensitization strategy is crucial for initiating appropriate treatment [7]. Hu L et al. reported that the use of cascade chemo-PDT with ROS efficiently synergizing with PD-L1 antibodies could inhibit primary and distant 4T1 (mouse breast cancer) tumor growth [20]. O'Shaughnessy MJ et al. demonstrated that vascular-targeted PDT in combination with PD-1/PD-L1 therapy evoked local and system immune responses to regress primary renal tumors and prevent lung metastasis in mice [21]. Most notably, Santos LL et al. demonstrated the clinical success of PDT followed by nivolumab treatment in advanced head and neck squamous cell carcinoma, which has greatly increased our confidence in PDT combined treatment [22]. However, the relationship between PDT-induced HIF-1α and PD-L1 expression remains to be thoroughly investigated. In this study, we confirmed that PDT might significantly promote the expression of these two important factors in lung cancer tissue with a positive correlation.

In NSCLC, whether hypoxia/HIF-1 pathways are predictive of poor or good outcomes is subject to debate. High HIF-1α expression is regarded as an indicator of poor prognosis [23]. However, another study found no relationship between the HIF-1α level and the survival rate of NSCLC [24]. The conflicting results in previous studies may be explained in part by the differing expression status of HIF-1α at distinct stages or extent of lymph node involvement [24, 25]. We come to the conclusion that the presence of a high HIF-1α level indicates an unfavorable prognosis in patients with lymph node-negative disease but not in those with lymph node metastasis, which is consistent with the previous study [25]. Interestingly in our analysis, high HIF-1α expression was instead a protective factor of early-stage lung cancer, and this may require sufficient and detailed data for further analysis. Despite these contradictory findings, hypoxia/HIF-1 pathways could be identified as key pathways affecting disease progression in NSCLC-PDT.

It has been shown that PD-L1 expression in tumors serves as a valuable prognostic factor, meaning that high level of PD-L1 is associated with improved immunotherapy outcomes [26, 27]. Many NSCLC patients fail to respond to PD-1 or PD-L1 therapy, primarily because of the poor immunogenicity and low tumor PD-L1 expression [7]. Clinical evidence has indicated that PDT stimulates the tumor-specific immune response, and may enhance ICIs efficacy [28]. Unfortunately, these findings are not clearly elucidated, especially when it comes to traditional PDT procedures. We employed bioinformatics analysis to obtain the critical pathways and genes to identify molecular markers relating to the outcome of immunotherapy in NSCLC-PDT, and found HIF-1α to be a promising key gene closely correlating with PD-L1 in HIF-1 and PD-L1-related pathways. In the following in-vivo experiments, PD-L1 and HIF-1α expression was shown to be upregulated by PDT in a strong consistent correlation. In short, the above data indicate that traditional PDT regulates HIF-1α/PD-L1 in NSCLC and thus might lead to a synergistic effect on immunotherapy.

HIF-1α is the key regulator of hypoxia induced apoptosis via HIF-1 pathway [29]. It could modulate Bcl-2 family members such as the anti-apoptotic protein Bcl-2 and proapoptotic protein Bax to promote apoptosis in tumors [11]. In this study, PDT activates HIF-1 pathway by inducing the key molecule HIF-1α expression, and promotes Bax and Bcl-2 recruitment as classical downstream targets along the HIF-1 pathway, while facilitating the emerging target PD-L1. Tunnel detection further demonstrated proapoptotic effect in PDT. These all indicate the important role of the HIF-1 pathway in apoptosis mechanism and immune effects in PDT.

Several theoretical and practical studies on the light dose impact of HIF-1α/PD-L1 expression are inconsistent. Wan J et al. believed that PDT-induced HIF-1α level in the low-dose group was higher than that in the high-dose group [30]. Gomer CJ et al. indicated that the higher the light dose, the more HIF-1α is expressed [31]. However, excessively low or high energy may lead to adverse outcomes. The 300 and 400 J/cm^2^ groups were the moderate dose groups with the best anti-tumor efficacy in this study. In the residual tumor lesions after treatment in the 300 and 400 J/cm^2^ groups, HIF-1α/PD-L1 expression was increased, indicating possible activation of tumor immune escape mechanisms. Theoretically, the residual disease with high PD-L1 expression can then be recognized and killed by subsequent immunotherapy.

On the one hand, the focal points of this study lie primarily on the following aspects: (1) the bioinformatics experiment in this study was relatively comprehensive and involved more than three network databases and hundreds of samples. (2) A relatively efficient and credible connection between PDT and immunotherapy for NSCLC was established, which provided good prospects for PDT research. (3) This paper innovatively proposes that traditional PDT might have synergistic anti-tumor effects by regulating the level of HIF-1α/PD-L1 in tumors when combined with immunotherapy.

On the other hand, our experiment still has some limitations. The xenograft nude mice model does not have a mature immune system, and is not an ideal model for building a complete tumor microenvironment. However, the goal of the existing work is to inspire future studies aiming to enhance the immune efficacy of treating.

NSCLC. (1) Focusing on tumor treatment: investigation of the mechanisms and optimal timing of combining PDT and ICIs is under way, and exploration of the impact of PDT regulating EGFR expression in lung cancer through the HIF-1 pathway on targeted therapy also holds promise [32]. (2) Focusing on molecular mechanisms: moreover, the crosstalk of HIF-1 and nuclear factor κB signaling pathways of PDT that can both affect molecular expression and immunologic function in hypoxia is thought-provoking [33]; the distinct regulation of HIF-1 or PD-1/PD-L1 pathway in pulmonary fibrogenesis and carcinogenesis deserves attention, which may contribute to the development of individual manangment in interstitial lung disease and lung cancer [34, 35].

## Conclusions

High HIF-1α/PD-L1 expression levels were found in NSCLC tissue after traditional PDT, which may assist in predicting the prognosis of NSCLC patients treated by PDT and PD-1/PD-L1 therapy. These results provide insight into the immune mechanism of PDT, as well as new concepts for respiratory intervention therapies and synergistic combination therapies for NSCLC.

## Data Availability

The data that support the findings of this study are available from the corresponding author upon reasonable request.

## Abbreviations

Bax: BCL2-associated X
BCA: Bicinchoninic acid
Bcl-2: B-cell lymphoma-2
BP: Biological process
CC: Cellular component
Ct: Comparative cycle
DCB: Durable clinical benefit
DEG: Differentially expressed gene
DNA: Deoxyribonucleic acid
EGFR: Epidermal growth factor receptor
FoxO: Forkhead box protein O
GEO: Gene Expression Omnibus
GO: Gene ontology
GSEA: Gene Set Enrichment Analysis
HIF-1α: Hypoxia inducible factor-1α
HPD: Hematoporphyrin derivative
ICI: Immune checkpoint inhibitor
IL: Interleukin
IQR: Interquartile range
KEGG: Kyoto Encyclopedia of Genes and Genomes
KM: Kaplan–Meier
MF: Molecular function
NDB: Non-durable benefit
NF-κB: Nuclear factor kappa-B
NSCLC: Non-small cell lung cancer
PARP1: Poly ADP-ribose polymerase 1
PBMC: Peripheral blood mononuclear cell
PD: Progressive disease
PD-1: Programmed cell death protein 1
PD-L1: Programmed death ligand 1
PDT: Photodynamic therapy
PIK3R1: Phosphoinositide- 3-kinase, regulatory subunit 1
PPI: Protein–protein interaction
PR: Partial response
RT-qPCR: Real-time quantitative reverse transcription polymerase chain reaction
RIPA: Radio immunoprecipitation assay
RNA: Ribonucleic acid
ROS: Reactive oxygen species
SD: Standard deviation
SDS‒PAGE: Sodium dodecyl sulphate–polyacrylamide gel electrophoresis
STAT: Signal transducer and activator of transcription
TBST: Tris-Buffered Saline Tween-20
TCGA: The Cancer Genome Atlas
TNF-α: Tumor necrosis factor α
TRIzol: Total RNA extraction reagent
TV: Tumor volume
